# Emotion regulation as a mediator on the relationship between emotional awareness and depression in elementary school students

**DOI:** 10.3389/fpsyg.2023.1127246

**Published:** 2023-03-15

**Authors:** Ruian Wang, Haoyue Li, Biao Sang, Yuyang Zhao

**Affiliations:** ^1^School of Psychology and Cognitive Science, East China Normal University, Shanghai, China; ^2^Department of School Counseling, Caoguangbiao Primary School, Shanghai, China; ^3^Lab for Educational Big Data and Policymaking, Shanghai Academy of Educational Science, Shanghai, China; ^4^Department of Social Work, School of Sociology and Political Science, Shanghai University, Shanghai, China

**Keywords:** emotional awareness, emotional intelligence, depression, emotion regulation strategy, elementary school children

## Abstract

As a cognitive skill, emotional awareness plays a fundamental role in emotional intelligence and significant effect on the development of individuals’ social adaptation. However, the role of emotional awareness in children’s social adaptation, especially emotional development, remains unclear, the current study sought to determine the significant influence of emotional awareness in children’s emotional development. By using cross-sectional and longitudinal study designs, the current study explored the relationship between emotional awareness and children’s depression, as well as the mediation effect of emotion regulation on this relationship. The sample comprised 166 Chinese elementary school students (89 girls and 77 boys) ranging from 8 to 12 years old. After adjusting for demographic variables (gender, grade, etc.), the results showed that children with high emotional awareness were less likely to adopt expressive suppression as an emotion regulation strategy and had lower depression levels currently and in the future. In contrast, children with low emotional awareness were more likely to use suppression strategies and showed higher depression levels. Thus, the results indicated that emotional awareness could predict children’s current and future depression status. Meanwhile, emotional regulation strategies are an important mediating variable explaining the relationship between emotional awareness and children’s depression. Implications and limitations were also discussed.

## Introduction

Depression is one of the most common psychological issues among children and adolescents. Its prevalence increases rapidly during puberty (Nolen-Hoeksema, 2002), leading to feelings of sadness, frustration, and helplessness; loss of interest in most activities; and interference with sleep, appetite, concentration, and experiences (Beck, 2005). As a complex compound emotion, depression is dominated by painful experiences that are stronger and more persistent than any single negative emotional experience (Sang and Liu, 2022). The World Health Organization (WHO) estimates that 50% of human mental health issues are experienced by children and adolescents, for whom depression is the leading cause of illness or disability (World Health Organization, 2019).

The *Report on Development of Youths in China*, released at the end of 2019, stated that approximately 30% of Chinese children and adolescents (under the age of 17) have experienced depressive symptoms and that such symptoms tended to present at a young age. Meanwhile, the *Report on National Mental Health Development in China (2019–2020)* stated that the detection rates of depression and severe depression in elementary school students were approximately 10% and 1.9–3.3%, respectively (Fu et al., 2021). As a developmental emotional issue, depression is—or has become—a major threat to the healthy growth and development of children and adolescents. Numerous studies have concluded that it impairs mental health and social functioning and increases the likelihood of committing suicide (Kessler and Bromet, 2013).

The causes of depression in children and adolescents are extremely complex. Mental health in children and adolescents can be affected by factors including poor parenting, the family environment, and academic and peer pressure. Because childhood is a sensitive period of growth, emotional problems are especially likely to cast psychological shadows that may be difficult to break free from throughout life. However, scholars have reported that depression is strongly connected to children’s emotional intelligence (EI) (Nejad and Nejad, 2012; Yang, 2012; Wang et al., 2013; Martínez-Monteagudo et al., 2019). This study sought to identify the elements of EI that impact the severity of depression among children. As detailed below, only a few studies have been conducted on this topic, and none have investigated how the interactions between different elements of EI affect depression in children.

### Depression in children and EI

EI, a theoretical framework originally proposed by Mayer and Salovey (1997), is a series of interrelated skills that enable individuals to perceive, understand, use, and regulate emotional events in an effective and adaptive way. They considered that EI should be the ability to (1) perceive, evaluate, and express emotions with precision; (2) access or generate thought-provoking emotions; (3) understand emotions and emotional information; and (4) regulate emotions for emotional and intellectual development (Mayer and Salovey, 1997). Following the successive proposal of multiple EI concepts, they subsequently simplified the construct into four main aspects (Mayer et al., 2000), namely: the capacities for (i) emotional awareness (EA) and expression, (ii) emotions that promote thinking, (iii) understanding and analyzing emotions, and (iv) regulating emotions. Emotion regulation (ER) refers to an individual’s ability to regulate their own emotions and those of others effectively, which is predicated on an awareness of emotional events. Monitoring and reflecting on ER facilitates emotional development.

These four aspects are closely related to particular variables that profoundly affect an individual’s abilities in areas such as emotional and social adaptations (Martínez-Monteagudo et al., 2019), and are inextricably connected with developmental emotional issues, such as depression. For example, individuals with low EI have difficulties obtaining social support to relieve the psychological stresses that they experience in life; this is compounded by the fact that they also tend to ruminate and have weak psychological resilience (Armstrong et al., 2011). This lack of adaptive ER makes them more likely to experience negative emotions such as anxiety and depression (Yang, 2012). Studies have shown that individuals with low EI score higher in anxiety and depression scales (Martínez-Monteagudo et al., 2019). Children with severe depressive symptoms are also more inclined to process and understand emotional information negatively or pay excessive attention to their own emotions (Gross, 2007). Furthermore, individuals with mood disorders face a range of issues when coping with emotional manifestations, such as incorrect understanding of emotions, negative reactions to emotions, and greater difficulties with emotional recovery (Gross, 2007).

### EA, ER strategies, and EI

Mayer and Salovey (1997) defined the perception and management of emotions as parallel branches of EI. In reality, EA is a cognitive ability within the various structures of EI. According to Gross (1998), the attention component of ER (which is applicable to emotional situations) reflects an individual’s ability to perceive emotions. However, some researchers have argued that EA is an independent and important foundational component of EI. In Goleman’s opinion, EI models share a common core that represents the ability to recognize and regulate ones’ emotions and those of others at a general level (Wang et al., 2013). Davies et al. (1998) even suggested replacing the concept of EI with EA. Nevertheless, as the field of EA continues to advance, researchers have gradually reached the consensus that it is a critical foundation and prerequisite for EI (Lane, 2000).

EA is not a novel concept without an origin. Rather, it derived from a deficit of EA in clinical practice and, relatedly, from recognition of the prevalence of alexithymia (Deng et al., 2013). Lane and Schwartz (1987) presented a cognitive-developmental theory of EA and described it as the ability to recognize and describe one’s and others’ emotions (Lane and Schwartz, 1987; Lane and Smith, 2021). Lane et al. (1990) compared the developmental stages of EA to Piaget’s cognitive development model and proposed a five-level EA developmental model. These levels, from low to high, comprise somatic responses; behavioral tendencies; and singular, mixed, and compounded complex emotions. The bottom two levels are implicit responses, while the top three levels are explicit responses.

ER is an important component of EI structures and involves the individual’s awareness of their emotions and how to express them (Wang et al., 2007). A key component in this process is the individual’s ability to perceive and describe the emotions they are experiencing. The ability to recognize emotions is related to EA; therefore, researchers have proposed that EA is an important basis for other components of EI, such as ER (Salovey et al., 2003). The ER process model proposed by Gross (1998) has been the most influential regarding the application of ER and the classification of ER strategies. Specifically, ER strategies in the situational, attention, evaluation, and reaction stages of Gross’s model include the selection and modification of a situation, the allocation of attention, cognitive reappraisal, and expressive suppression. Previous studies have found that cognitive reappraisal and expressive suppression have the most significant effects on the ER process among the strategies (Gross et al., 2006); therefore, the present study focused on these two strategies.

The development of ER skills is generally considered an achievement. Children typically learn how to express and regulate their emotions gradually as they age. However, those who experience internalization–externalization issues during the developmental process may demonstrate a relative lack of EA abilities, which may, in turn, affect their adaptive ER (Stegge and Terwogt, 2007). Specifically, individuals with ER issues tend to adopt maladaptive ER strategies because cognitive reappraisal is more difficult than expressive suppression because it requires additional emotional experiences and information (Gross, 1998; Sheppes et al., 2011).

Based on this existing knowledge, we proposed that individuals deal with emotions in two steps characterized by EA and ER. These steps stress the spontaneous nature of the emotional process and emphasize the need to regulate emotions. In the first step, the individual must analyze their situation and comprehend the nature of the problem before deciding on the optimal response (EA). In the second step, they must set goals and make and evaluate strategic choices (ER) (Gross, 2007). EA is not merely an incidental phenomenon; it helps individuals participate in automated control activities (ER) that facilitate adaptive behaviors (Levenson, 1999). In other words, children must scrutinize their emotions, adjust their attention carefully, and, finally, use their emotions proficiently to gain adaptive skills to regulate their emotions adequately. Therefore, we ultimately proposed that EA is the foundation of EI and that using ER concretely embodies EI.

### Relationship between EA, ER strategies, and depression

Researchers have argued that EA involves paying attention to and reflecting on your somatic responses to your emotions. Individuals use EA to extract intrinsic emotional information, infer the meaning of the current interpersonal interaction and related situational needs (Greenberg, 2016), and determine whether ER is necessary. With advancements in EA-related research, researchers have gradually recognized that it comprises an essential element of ER and mental health (Barrett and Gross, 2001). Individuals with poor EA experience emotional instability because they cannot adequately recognize emotions (Thompson et al., 2009). Consequently, they frequently experience related physical symptoms (Lane et al., 2011) and self-doubt (Boden and Berenbaum, 2007). Emotional adaptation issues, such as social anxiety (Charry et al., 2004) and depression (Klemanski et al., 2017), easily emerge in this context.

Researchers have further proposed that EA may be a critical factor in mood swings during adolescence (Somerville et al., 2010). EA has demonstrated a negative correlation with depression during adolescence (Rieffe and De Rooij, 2012; Sendzik et al., 2017). Some studies involving children and adolescents found that poorer EA was often associated with a higher prevalence of clinical depressive symptoms (Klemanski et al., 2017). For example, a survey of 356 Chinese children in grades 4–6 found that EA was significantly negatively correlated with depression and anxiety (Gao, 2016). The baseline level of EA abilities could even be used to predict children’s depression level 1 year later (Kranzler et al., 2016). In sum, literature suggests that children’s EA abilities substantially affect their depression.

Numerous studies on children’s ER strategies and depression suggest that the former is an important factor affecting the latter. Children with good ER can flexibly use various strategies in relation to their environment. A negative correlation between ER and negative emotions has also been identified (Eisenberg et al., 2007). Early studies examined individuals who applied cognitive reappraisal and expressive suppression in specific situations and found that these two strategies were negatively and positively correlated with depression and other psychological disorders, respectively (Aldao et al., 2010). Accordingly, cognitive reappraisal is more adaptive than expressive suppression (Webb et al., 2012). Therefore, cognitive reappraisal is generally regarded as an adaptive strategy and expressive suppression as a non-adaptive strategy.

Meanwhile, researchers have found that children with good ER respond more empathically and are more likely to practice adaptive regulation strategies, such as cognitive reappraisal. In contrast, individuals with poor ER frequently use expressive suppression and demonstrate emotional maladjustment (Chervonsky and Hunt, 2017). A meta-analysis of 35 studies highlighted that adolescents’ choices of adaptive ER strategies (e.g., cognitive reappraisal) were significantly negatively correlated with their depressive symptoms, whereas their choices of non-adaptive ER strategies (e.g., expressive suppression) were significantly positively correlated with depressive symptoms (Schäfer et al., 2017).

From the perspective of Beck’s (2005) cognitive theory of depression, depressed individuals form a negative cognitive schema based on their genetics and early traumatic experiences, causing them to develop depressive beliefs. These include viewing themselves, the world around them, and the future pessimistically. The schema is activated by situations with a similar emotional valence. During subsequent information processing, the resultant pessimistic thoughts strengthen the individuals’ negative cognitive biases through the attention and evaluation that the individuals assign to them (Beck and Bredemeier, 2016). Existing studies have confirmed that rather than being sensitive to emotional stimuli, depressed individuals pay attention to negative emotional stimuli when attempting to resolve their difficulties, resulting in sustained processing of the stimuli (Joormann and D’Avanzato, 2014). Thus, depressed individuals tend to become immersed in their identified negative emotions and then automatically and persistently process information in a non-adaptive way to deal with them. Studies have found that patients with depression showed more obvious symptoms of alexithymia, that is, having difficulty recognizing and describing their own emotions (e.g., Zhang et al., 2017), while ER ability may be an independent trait with no connection to the depressive state. Depression appears to have greater effects on EA than ER. However, studies (e.g., Zhang et al., 2022) have pointed out obvious differences between depressed individuals and ordinary people during their selection of ER strategies, which are affected by factors such as emotional valence and intensity.

As established above, numerous studies have explored the impact of EA on promoting ER, the significance of using ER strategies to manage depression, and the effect of depression on EA and ER. However, there is a lack of research on the relationship between EA, ER, and depression. Although scholars have suggested that these variables are closely related, the functions of the variables in EI remain unclear. In response to this gap in the existing literature, we asked: how is EA (as a cognitive ability) related to ER and emotional development issues (e.g., depression) in children?

According to researchers, EA determines whether ER strategies are successful. In turn, the use of a specific ER strategy affects the outcome of an emotional experience and response. The habitual use of maladaptive ER strategies eventually leads to physical and mental illnesses (Van Beveren et al., 2019). When individuals experience negative emotions, their EA can help them choose adaptive regulation strategies, thereby effectively alleviating these emotions. Conversely, a lack of EA can lead to maladaptive behaviors and persistent negative emotions that cause subsequent issues. Accordingly, we reasoned that ER is involved in the relationship between maladaptation and EA (Barrett and Gross, 2001) and that individuals must thus recognize and understand their emotions when choosing an ER strategy and deciding on the optimal solution.

Some researchers have hypothesized that EA affects depression through ER. Specifically, their findings indicated that individuals who paid more attention to emotional information and could clearly recognize their emotions were more inclined to choose cognitive reappraisal as a strategy, thereby facing fewer emotional adjustment issues (e.g., depression) (Boden and Thompson, 2015). In other words, individuals with high EA considered cognitive reappraisal more effective and used it for ER, thereby avoiding depressive symptoms. However, individuals who paid less attention to, and tended to be confused by, their emotions were more likely to use expressive suppression, resulting in a greater prevalence of depression (Boden and Thompson, 2015). In a study of adolescents, Vine and Aldao (2014) proposed that adverse ER strategies had an obvious mediating effect in the relationship between EA and emotional issues. Poor EA can be a major contributor to the development of depressive symptoms during adolescence if individuals are not adept at using adaptive ER strategies because additional cognitive resources (i.e., good EA) are required to activate such strategies; non-adaptive strategies are automatic and somatic (Van Beveren et al., 2019).

### The present study and hypotheses development

Although there is no related empirical research involving children, we deduced from existing research that a lack of EA was related to insufficient ER (Hubbard et al., 2002; Stegge and Terwogt, 2007; Eckland and English, 2019) and non-adaptive ER strategies. These tendencies caused emotional adjustment issues (Eisenberg et al., 2007; Blair et al., 2015), such as childhood depression. Accordingly, we reasoned that a mediating relationship exists between children’s EA, ER, and emotional adaptation. However, there is a lack of longitudinal follow-up research on the mechanism by which the various variables interact in child development. Most existing studies on the topic are cross-sectional (Sendzik et al., 2017), and the mutual interactions between the variables during the developmental process are yet to be examined. Thus, the predictive mechanism of EA, ER, and emotional adaptation during child development remains unclear.

Children aged 8–12 are just beginning the transition from childhood to adolescence. They require access to additional ER strategies as they enter the middle-upper grades of elementary school, and the significance of emotional adaptation should become more apparent to them during this time. Meanwhile, lack of EA, which also affects the preference of ER strategies, might become an important risk factor in their future. Thus, it is necessary to investigate their psychological wellbeing by analyzing their EA and depression, especially since children of these ages have accumulated sufficient language skills, emotional knowledge, and social experience.

Therefore, we conducted a cross-sectional study as well as a longitudinal study and used a mediation model to examine the role of EA on children’s depression and the significance of ER strategies in this relationship. Ultimately, we sought to determine the significance of EA in children’s emotional development and how that significance was produced.

Based on the above, this study hypothesized that EA could predict children’s current and future depression levels through ER strategies. Specifically, children’s EA abilities were positively and negatively correlated with the application of the cognitive reappraisal and expressive suppression strategies, respectively, while ER strategies had a mediating effect between EA and depression.

We hoped that this study would reveal the impact of the key elements of EI (i.e., EA and ER) on children’s depression, as this would assist in finding the right approach to improving school counseling.

## Materials and methods

### Participants

All of the participants in this study were children from mainland China, followed over 3 years from grades 3–5. The children were recruited from 47 classes in three elementary schools in Shanghai *via* random sampling.

Before conducting the study, the researchers administered the Chinese revision of the Levels of Emotional Awareness Scale for Children (LEAS-C; Wang et al., 2015) to 855 students in grades 3–5 (*M*_age_ = 9.57, SD = 0.89) to detect differences in ER strategies and depression levels among children with varying EA levels. After referring to previous studies, the top and bottom 27% of participants, based on their total scores for the scale, were used to distinguish between those with high and low EA, respectively (Kelley, 1939). In this study, children with a total score of ≥41.5 on the EA questionnaire were screened as having high EA; those with a total score of **≤**33 were considered as having low EA. The final sample comprised 166 children who met the requirement for random selection and voluntarily agreed to long-termparticipation (Table 1).

**Table 1:** The demographic variables of the participants in the two groups.

	High EA group (82)	Low EA group (84)	Total (166)
Male	40	37	77
Female	42	47	49
*M*_age_	9.62	9.66	9.59
SD	0.78	0.90	0.84
Only child	37.80%	48.81%	44%
Fathers completed high school or above	54.88%	71.19%	68.07%
Mothers completed high school or above	60.98%	67.86%	66.05%

The results of the independent sample *t* test indicated a significant difference between the high and low EA groups in terms of their total LEAS-C scores, *t* (164) = 21.97, *p* < 0.001, Cohen’s *d* = 3.43. The score of the high EA group for the questionnaire was *M* = 43.30, *SD* = 1.55; that of the low EA group was *M* = 20.39, *SD* = 9.32.

### Measures

#### The levels of emotional awareness scale for children

To measures levels of EA, children completed the Chinese revision of the LEAS-C (Wang et al., 2015), which was developed by Bajgar et al. (2005). This scale plots 12 scenarios, each based on two people (the self and others) to elicit one of the four types of emotions (anger, fear, happiness, and sadness). For each scenario (e.g., “Someone who used to criticize you gave you a compliment”), two questions are posed “How do you feel?” and “How do you think the other person feels?” The scorers (at least two) then rate the participants’ responses. Three scores are allocated for each scenario, one each for self-awareness, other-awareness, and total awareness. The total awareness score is the higher of the scores. Each scenario is rated on a 5-point scale. Ratings for each scenario are summed to give a maximum possible score out of 60. Higher scores indicate a higher level of EA in a dimension or overall. In the current sample, internal consistency for self-awareness, other-awareness, and total awareness was 0.95, 0.97, and.93, respectively.

### Emotion regulation questionnaire for children and adolescents

The Chinese version of the Emotion Regulation Questionnaire for Children and Adolescents (ERQ-CA-C; Chen et al., 2016), which was initially developed by Gullone and Taffe (2012), was used. This questionnaire aims to measure the tendency of children and adolescents to use two ER strategies: expressive suppression and cognitive reappraisal. The questionnaire consists of 10 items. Cognitive appraisal strategies (e.g., “When I want to be happy, I think of something else, such as my teacher praising me today”) are calculated by averaging the scores of items 1, 3, 5, 7, 8, and 10. Expressive suppression strategies (e.g., “When I am happy, I am careful not to show it”) are calculated by averaging the scores of items 2, 4, 6, and 9. Items are rated on a 5-point Likert scale, ranging from “completely disagree” (1) to “completely agree” (5). Higher scores indicate a higher tendency to use a particular strategy. Cronbach’s *α* for the cognitive reappraisal and expressive suppression dimensions was 0.76 and 0.44, respectively. Since the expressive suppression dimension had only four questions and the participants in this study were from the high and low EA groups, a relatively low internal consistency coefficient was acceptable.

### Children’s depression inventory

This study adopted the Chinese version of the Children’s Depression Inventory (CDI) scale, developed by Kovacs (1992) and revised by Yu and Li (2000). The scale consists of 13 items measuring children’s depression-related feelings, thoughts, and behaviors. Each item described three levels of depressive symptoms (e.g., “I am occasionally unhappy,” “I am often unhappy,” and “I am always unhappy”), and participants are asked to choose the one that best fits their actual situation. After reverse-scoring items 2, 5, 6, 7, 12, and 13, the mean score is calculated, with higher scores indicating higher levels of depression. This questionnaire has been shown to have good reliability and validity (Yu and Li, 2000; Wu et al., 2010; Liu et al., 2015). Cronbach’s *α* of this questionnaire were 0.90 and 0.90, respectively, for the two measurements of the study.

## Design and procedure

Before the survey began, informed consent was obtained from each school principal, classroom teacher, and parents of participating students, as well as from the students themselves. In the survey, a single class was taken as a unit and students completed the questionnaires with paper and pencil. The experimenters were graduate students trained in developmental and educational psychology, as well as psychology teachers, and head teachers. Before each test was administered, participants were informed that they were required to answer questions faithfully, with no right or wrong answers, and the principle of confidentiality would be strictly observed.

This survey was conducted twice, 6 months apart, to collect the longitudinal data. The first survey was conducted in the first semester of the 3rd, 4th, and 5th grades (T1 in mid-October of the current year), measuring EA, ER strategies, and children’s depression levels. The second survey was conducted in the second semester of the 3rd, 4th, and 5th grades (T2 in mid-May of the following year), and measured children’s depression levels. No participants dropped out or were added between the two tests.

The participants were required to complete the LEAS-C, ERQ-CA-C, and CDI assessments at T1, and the CDI at T2. The questionnaires were administered in class and collected immediately upon completion, which took about 30 min and 15 min at T1 and T2, respectively. The LEAS-C questionnaires were collected and scored by two research assistants according to the scoring manual, and the final score for each item and dimension of the individual was calculated by averaging the scores of the two raters.

### Data analysis

Descriptive statistical analysis was performed using SPSS 26.0. The bootstrap method of the PROCESS model 4 macro program, developed by Hayes (2013) in SPSS, was used to examine the mediating role of ER strategies between EA and child depression. A mediating effect test was run with 5000 resamples and a 95% confidence interval. If the 95% confident interval for the mediating effect did not contain 0, the effect was considered significant.

## Results

### Descriptive analyses

The mean and standard deviation of the children’s EA, different ER strategies, and level of depression at T1 and T2 are displayed in Table 2.

**Table 2:** The mean and standard deviation of the main study variables.

	High EA group		Low EA group	
	*M*	*SD*	*M*	*SD*
Total awareness (T1)	43.30	1.55	20.39	9.32
Expressive suppression (T1)	2.89	0.81	3.29	0.89
Cognitive reappraisal (T1)	3.69	0.96	3.63	0.86
Depression (T1)	1.37	0.36	1.51	0.49
Depression (T2)	1.33	0.37	1.56	0.44

Expressive suppression strategies and EA ability (high and low scores) were adopted as the dependent and independent variables, respectively. The control variables were demographic variables including the children’s gender and grade, parents’ educational level, and status of being an only child or not. Analysis of variance (ANOVA) was performed; the results showed that EA had a significant main effect *F* (1, 145) = 9.67, *p* = 0.00, *η^2^* = 0.06. The *post-hoc* test results revealed that children with high EA had significantly lower expressive suppression scores than those with low EA.

Next, ANOVA was performed using cognitive reappraisal strategies and EA abilities (high and low scores) as the dependent and independent variables, respectively, and the demographic variables comprised the control variables. The results showed that the main effect of EA was not significant *F* (1, 145) = 0.07, *p* = 0.80, *η^2^* = 0.00. There was no difference between the children in the high and low EA groups in terms of their cognitive reappraisal scores.

A 2 × 2 repeated ANOVA was conducted with depression as the dependent variable, measurement time as the within-subject independent variable (T1, T2), EA (high and low scores) as the between-group independent variable, and demographic variables as the control variables. The results indicated that the between-group main effect was significant *F* (1, 145) = 7.57, *p* = 0.01, *η^2^* = 0.05. The *post-hoc* test showed that children in the high EA group had significantly lower scores on the depression scale than those in the low EA group. The main effect of measurement time was not significant *F*(1, 145) = 1.61, *p* = 0.21, *η^2^* = 0.01, nor was the interaction effect between measurement time and EA *F*(1, 145) = 1.44, *p* = 0.23, *η^2^* = 0.01. Therefore, the depression variable remained stable for both groups of participants over the two time points.

### Analysis of the mediating effect of emotion regulation strategies between emotional awareness and depression

#### Mediating model in a cross-sectional study

**Figure 1:**
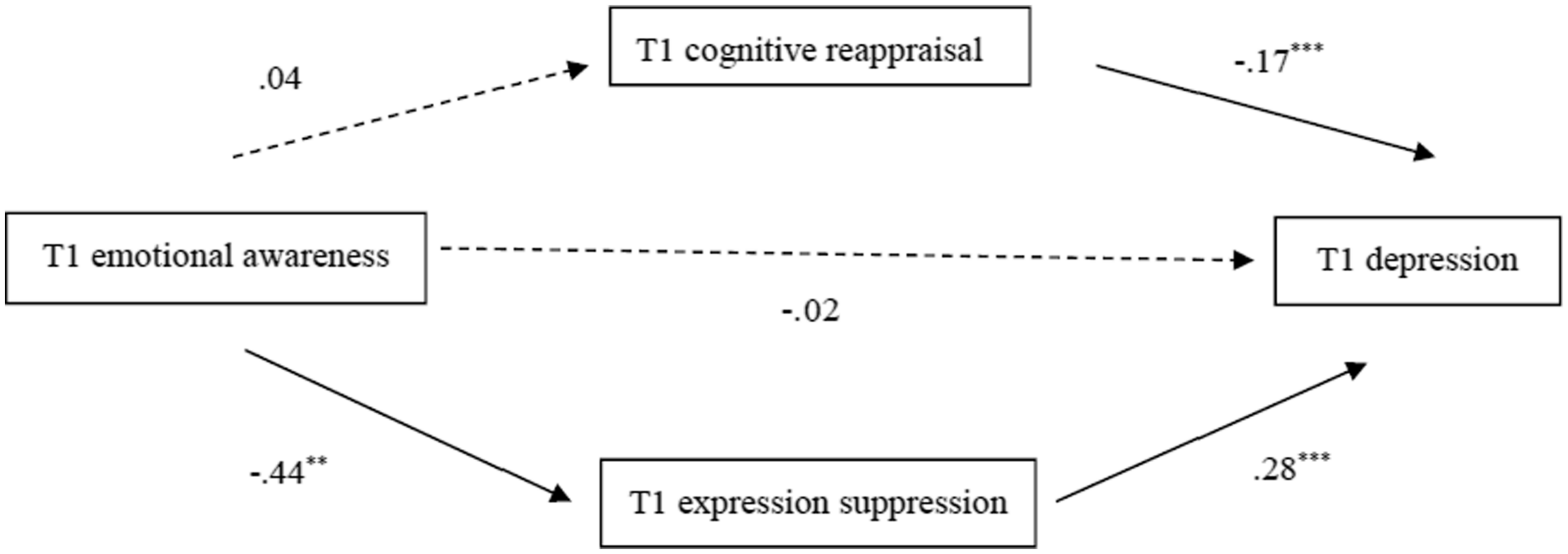
Mediating model of emotion regulation strategies between *T1 emotional awareness* and *T1 depression.* Straight lines represent the coefficient is significant. Dashed lines represent the coefficient is not significant. The coefficient from emotional awareness to depression below the center horizontal path represents the direct effect. **p < 0.01, ***p < 0.001.

Taking *T1 emotional awareness* as the independent variable and *T1 depression* as the dependent variable (dummy variables, 1 = high EA group, 0 = low EA group), the mediating effect of expressive suppression and cognitive reappraisal strategies was tested after controlling for child gender, grade, parental education, and only child status. The results showed that *T1 expressive suppression* played a mediating role between *T1 emotional awareness* and *T1 depression* (see Figure 1), with an indirect effect size of −0.13, 95% CI = [−0.225, −0.036], and a mediating effect of 80.40% of the total effect. That is, children with higher EA scores were less likely to adopt expressive suppression (*B* = −0.44, *SE* = 0.14, *p* < 0.01) and had lower levels of depression (*B* = 0.28, *SE* = 0.03, *p* < 0.001). The mediating effect of cognitive reappraisal was not significant (see Figure 1), 95% CI = [−0.063, 0.044] between EA and depression. EA was a predictor of cognitive reappraisal (*B* = 0.04, *SE* = 0.15, *p* = 0.80), and cognitive reappraisal was a negative predictor of children’s depression at T1 (*B* = −0.17, *SE* = 0.03, *p* < 0.001).

#### Mediating model in a longitudinal study

**Figure 2:**
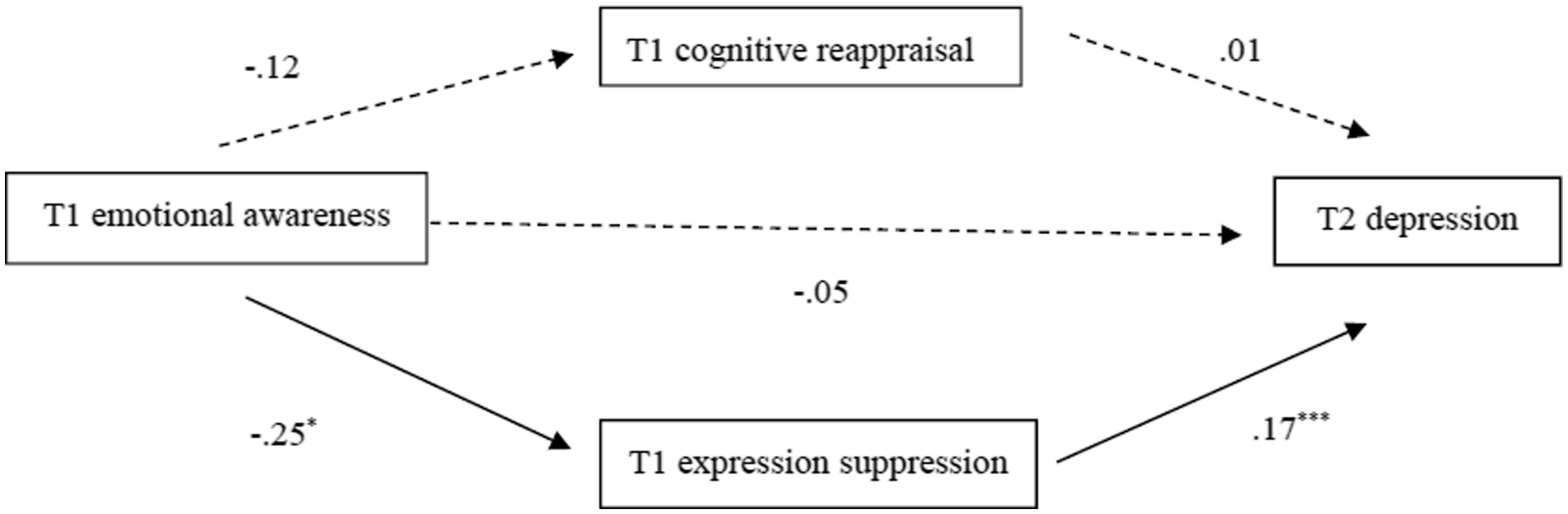
Mediating model for emotion regulation strategies between *T1 emotion awareness* and *T2 depression.* Straight lines represent the coefficient is significant. Dashed lines represent the coefficient is not significant. The coefficient from emotional awareness to depression below the center horizontal path represents the direct effect. *p < 0.05, ***p < 0.001.

Taking *T1 emotional awareness* as the independent variable and *T2 depression* as the dependent variable (dummy variables, 1 = high EA group, 0 = low EA group), the mediating effect of expressive suppression and cognitive reappraisal at T1 was tested after controlling for demographic variables and *T1 depression*. The results showed that *T1 expressive suppression* mediated *T1 emotional awareness* and *T2 depression* (see Figure 2), with an indirect effect size of −0.04, 95% CI = [−0.090, −0.002], and a mediating effect of 43.12% of the total effect. That is, children with higher EA scores were less likely to adopt expressive suppression (*B* = −0.25, *SE* = 0.11, *p* = 0.03) and subsequently had lower levels of depression at T2 (*B* = 0.17, *SE* = 0.03, *p* < 0.001). The mediating effect of cognitive reappraisal was not significant (see Figure 2), 95% CI = [−0.016, 0.007]. Thus, EA was not a predictor of cognitive reappraisal (*B* = −0.12, *SE* = 0.14, *p* = 0.40), and cognitive reappraisal was not able to predict children’s depression at T2 (*B* = 0.01, *SE* = 0.02, *p* = 0.59).

## Discussion

The results of this study were consistent with the research hypotheses. Specifically, first, in the cross-sectional study, ER strategies played a mediating role in the relationship between EA and children’s depression. Children with high EA were less likely to adopt ER strategies involving expressive suppression and had lower levels of depression. By comparison, children with low EA were more inclined to adopt suppressive strategies that led to higher levels of depression. Second, the longitudinal study verified the existence of the mediating effect that ER strategies had on EA and depression. Children with high EA were less likely to adopt ER strategies involving expressive suppression and thus had lower depression levels over time. In contrast, those with low EA tended to adopt expressive suppression strategies that led to higher depression levels over time.

Previous studies on EA and depression in children and adolescents are not uncommon (Somerville et al., 2010; Rieffe and De Rooij, 2012; Gao, 2016; Sendzik et al., 2017). Our cross-sectional findings demonstrated that EA could predict children’s depression through ER strategies. Our findings also confirmed that interactions between the three variables were long lasting. This is likely because children with poor EA tend to have insufficient ER abilities (Hubbard et al., 2002; Stegge and Terwogt, 2007; Eckland and English, 2019); moreover, non-adaptive ER or poor ER strategies lead to emotional maladjustment in children (Eisenberg et al., 2007; Blair et al., 2015).

The results of the longitudinal study not only demonstrated this reasonable speculation but, more importantly, supplemented the findings of previous studies. Also of critical value are the long-term results established in this study, which introduced a new perspective on depression in children and adolescents. The causes of depression are complex; ER has always been considered closely related to depression because the latter is a developmental emotional issue (Eisenberg et al., 2007; Aldao et al., 2010; Webb et al., 2012; Chervonsky and Hunt, 2017). However, this study confirmed that EA might affect the tendency to select specific ER strategies, thus allowing children’s future depression level to be predicted. Minimally, this revealed that children are affected by their own EA levels before regulating the emotions that they experience, and that this impact acts on their future depression levels. Specifically, EA was found to influence the children’s choice of expressive suppression strategies significantly, which, in turn, affected their depression. This was consistent with the assertions that (i) lack of EA leads to the tendency to employ maladaptive ER strategies (such as expressive suppression) (Hubbard et al., 2002) and (ii) real situations in which frequent use of expressive suppression as a regulation strategy were associated with depression (Schäfer et al., 2017). These findings pointed to the necessity of including EA in the group of factors that cause depression in children, as well as the formation of at least one action path, namely EA → ER strategy (expressive suppression) → depression in children.

In terms of specific ER strategies, this study found that the expressive suppression strategy had a mediating effect between EA and children’s depression, but could not establish whether cognitive reappraisal, an adaptive regulation strategy, played a mediating role between these two variables. This differed slightly from the findings of previous studies (i.e., Eastabrook et al., 2014; Subic-Wrana et al., 2014; Van Beveren et al., 2019). In fact, existing findings on the mediating role of cognitive reappraisal are inconsistent. For example, Van Beveren et al. (2019) found that this strategy played a mediating role between EA and depression in adolescents, but this effect was not identified by Boden and Thompson (2015). Although a lack of EA affects children’s ER to a certain extent (Hubbard et al., 2002), some researchers believe that there is no direct relationship between EA level and the tendency to use cognitive reappraisal. Individuals who apply this strategy may be unaffected by EA during their tasks (Szczygieł et al., 2012).

The results of existing cross-sectional studies showed a strong relationship between cognitive reappraisal and depression. In one such study of children aged 10–12, the researchers found that the employment of cognitive reappraisal strategies negatively predicted the level of depression: the more frequent the use of such strategies, the lower the level of depression (Gullone and Taffe, 2012). A similar result was found among adolescents: cognitive reappraisal was more closely associated with depression in individuals than expressive suppression (Schäfer et al., 2017). However, some studies suggested that the development of children’s ER strategies is influenced by their cognitive abilities and social experiences. Some researchers proposed that children do not learn to use cognitive methods to regulate their emotions until they are 8 or 9 years old (Garnefski et al., 2007), meaning that their use of cognitive reappraisal strategies is unstable before that age. For example, one study found that the propensity to use cognitive reappraisal changed with age: compared with elementary school children, adolescents in high school were significantly more inclined to use cognitive reappraisal (Jiang et al., 2008). In contrast, this study found that the use of expressive suppression remained stable and did not reflect differences with development over age (Jiang et al., 2008). Cognitive reappraisal could not effectively predict children’s future depression levels in this study. Other researchers pointed out that the use of ER strategies is related to the potency and intensity of the stimuli that individuals face. Individuals tend to employ cognitive reappraisal when faced with high-intensity negative stimuli, but not when faced with high- or low-intensity positive emotions or low-intensity negative emotions (Zhang et al., 2022).

In summary, the cognitive reappraisal strategy did not demonstrate a mediating effect in this study. There was also no difference between children with varying EA abilities in their tendencies to use that strategy; however, the strategy could predict children’s current depression levels in cross-sectional studies. This might be because the applications of the cognitive reappraisal strategy were not always affected by EA, as the children’s ER skills were still developing and they still lacked sufficient cognitive abilities to use it. Their application of cognitive reappraisal strategies was unstable at that age and thus, this variable was not always associated with depression. Alternatively, it could be because the children were not subjected to intensive negative emotional experiences during this study. Ultimately, this study revealed that the expressive suppression strategy played a mediating role between EA and children’s depression and that children with poor EA abilities were more inclined to adopt this strategy, which led to higher levels of depression.

## Implications

This study aimed to broaden knowledge about children’s current EA abilities and depression to offer insights useful to researchers, educators, and pediatric clinical psychologists. Specifically, our findings may help such professionals make timely adjustments to their educational goals, methods, and interventions.

At present, approximately 30 million children and adolescents under the age of 17 in China have suffered or are suffering from various emotional disorders and behavioral issues (Jiang and Lei, 2021), which may adversely affect their development. Unfortunately, parents and teachers presently lack highly effective treatment and intervention methods for children and adolescents suffering from depression; they cannot even detect abnormalities in children at an early stage and in a timely manner. Parents and teachers often only pay attention to these issues when the symptoms have developed to the point that they can no longer be ignored.

We believe that an important component of school and family education should be the cultivation of emotional adaptation in children and adolescents. Adults must help youth learn effective ER methods (e.g., increasing their conscious thinking about emotions and being able to view difficulties and negative events rationally) and actively intervene. Clinical studies involving adults have shown that the level of EA was an important variable for the effectiveness of clinical interventions (Beutel et al., 2013) and that there might be a strong correlation between EA and interventional effects (Lane et al., 2020).

However, intervening in the EA abilities of elementary school children alone will not necessarily improve their anxiety and depression (Gao, 2016); the relationship between EA and depression may be affected by other important factors, such as ER. This study’s identification of the EA → ER strategies → depression path establishes that the risk of emotional issues in children can be reduced by optimizing and improving their EA abilities. During follow-up research, it is necessary to further investigate the scheme for optimizing children’s EA and the use of adaptive ER strategies (i.e., cognitive reappraisal) by children with different EA abilities. Doing so will clarify the impact of EA on the choice of ER strategies and its interventional effects on emotional adaptation.

Additionally, this study established that EA not only predicted children’s current depression through ER strategies but also predicted their future depression levels. This may help professionals approach their work from multiple perspectives based on the specific path identified in this study and reduce mental health risks in children caused by insufficient EA. Professionals may also offer youth further supports to improve their emotional adjustment and ability to cope with stress. For example, they may develop relevant 6–8 week courses training students in EA (e.g., improving their ability to recognize expressions and being exposed to situations involving various emotions), change their cognitive evaluation through cognitive ER strategies, and regulate emotion-related behavioral tendencies to change maladaptive emotional behaviors (Gross, 2007).

As another example, the OECD proposed that learning social and emotional skills can help children face new challenges and adapt rapidly to a changing society (OECD, 2021). The oft-emphasized Social and Emotional Learning course includes important content such as EA, pressure management, and coping, and is being accepted by increasing numbers of schools. When students are taught social and emotional skills, the effect on the students is considerable (Durlak et al., 2011). The transition from childhood to adolescence is particularly sensitive to increases in social and emotional skills, which may shape children’s behavior and lifestyles to achieve better personal and professional outcomes (OECD, 2021).

Meanwhile, we hope to combine real cases from daily life in our future research to propose effective prevention and intervention strategies from multiple perspectives, including those of parents, teachers, students, and managers. This will help us apply our research findings to practice.

## Limitations

Despite the revelations and contributions of this study, some elements must be further explored in future studies to enrich research in the field of children’s EA and depression. First, there is a need to improve and develop an appropriate measurement tool, especially for children. The LEAS-C, which was applied in this study, undoubtedly posed a certain degree of difficulty for our participants when answering the questions. They had to report all their own and others’ emotional experiences in each situation either in writing or orally; accordingly, the questionnaire took them nearly 30 min to complete. Subsequently, multiple trained professionals were needed to score the questionnaires, each of which similarly took nearly 30 min. Some researchers have tried to improve the scale by adjusting the style of the questions, including more points for scoring (Veirman et al., 2016) or using computer programs for scoring (Barchard and Picker, 2018). However, they could not resolve the problems concerning completing and scoring the questionnaire, which were time-consuming and labor-intensive. There may also be negative effects on the scoring of the scale, especially when the participants are children (Veirman et al., 2016). This issue should be addressed in future studies.

Second, the ERQ-CA-C questionnaire used to measure children’s ER strategies in this study was based on the ER process model, which was developed based on the Emotion Regulation Questionnaire (ERQ) intended for adults. The ERQ examines the ER strategies commonly used by children and adolescents aged 10–18. However, it only contains two ER strategies: cognitive reappraisal and expressive suppression. The number of questionnaire items is also limited (with only four items for expressive suppression), which is not conducive to measuring the dimensions accurately. Moreover, it does not involve any specific situations for eliciting and regulating emotions. Additionally, some researchers proposed that children are unable to regulate their emotions through cognitive means until they are 8 or 9 years old (Garnefski et al., 2007). The participants of this study were 8 or older, and might have been in the “transitional zone” in terms of developing ER strategies; this could possibly lead to inaccurate measurements when using the questionnaire. In future research, it is necessary to develop an ER questionnaire suitable for children under 10 years old and involves additional everyday situations and ER strategies.

Third, future studies should use an appropriate number of samples and duration for data tracking. The sample size of this study was relatively small because of the Covid-19 pandemic, which made it challenging to track more participants off-line. Further, the data in this study were limited to students in grades 3–5 and only collected over two school terms. This was due to the limitations of the conditions, such as the complexity of filling out the questionnaire and students having graduated. With the grade 5 students about to finish elementary school and move on to middle school, it was difficult to track their data over a third school term to analyze developmental trends or draw more convincing developmental conclusions. In the future, researchers may start data tracking earlier or select samples of students from schools offering 9-year compulsory education.

## Conclusion

This study found that EA negatively predicts current and future levels of depression in children. Unlike children with low EA, those with high EA were less likely to adopt ER strategies involving expressive suppression. In turn, they demonstrated lower levels of depression and were more likely to continue to demonstrate lower levels of depression in the future. Based on these results, we proposed the following possible path for using EA to predict children’s current and future mental health: EA → ER strategies → depression. This offers a new perspective useful for intervention in clinical and educational practice.

## Data availability statement

The original contributions presented in the study are included in the article/supplementary material, further inquiries can be directed to the corresponding authors.

## Ethics statement

The studies involving human participants were reviewed and approved by University Committee on Human Research Protection, East China Normal University, China. Written informed consent to participate in this study was provided by the participants’ legal guardian/next of kin.

## Author contributions

RW contributed to the design of the work, data collection, drafting the article, and its critical revision. HL contributed to the data analysis and interpretation. BS and YZ contributed to the final approval of the version to be published. All authors provided critical feedback and shaped the manuscript.

## Funding

The current study was funded by Shanghai Academy.

## Conflict of interest

The authors declare that the research was conducted in the absence of any commercial or financial relationships that could be construed as a potential conflict of interest.

## Publisher’s note

All claims expressed in this article are solely those of the authors and do not necessarily represent those of their affiliated organizations, or those of the publisher, the editors and the reviewers. Any product that may be evaluated in this article, or claim that may be made by its manufacturer, is not guaranteed or endorsed by the publisher.
